# CIP2A regulates MYC translation (via its 5′UTR) in colorectal cancer

**DOI:** 10.1007/s00384-020-03772-y

**Published:** 2020-10-19

**Authors:** S. Denk, S. Schmidt, Y. Schurr, G. Schwarz, F. Schote, M. Diefenbacher, C. Armendariz, F. Dejure, M. Eilers, Armin Wiegering

**Affiliations:** 1 Department of Biochemistry and Molecular Biology, Biocenter, University of Würzburg, Würzburg, Germany; 2 Department of General, Visceral, Transplant, Vascular and Pediatric Surgery (Department of Surgery I), University Hospital Würzburg, Oberduerrbacherstr. 6, 97080 Würzburg, Germany; 3 Comprehensive Cancer Center Mainfranken, University of Würzburg, Würzburg, Germany

**Keywords:** CIP2A, MYC, Translation, Colon cancer

## Abstract

**Background:**

Deregulated expression of MYC is a driver of colorectal carcinogenesis, suggesting that decreasing MYC expression may have significant therapeutic value. CIP2A is an oncogenic factor that regulates MYC expression. CIP2A is overexpressed in colorectal cancer (CRC), and its expression levels are an independent marker for long-term outcome of CRC. Previous studies suggested that CIP2A controls MYC protein expression on a post-transcriptional level.

**Methods:**

To determine the mechanism by which CIP2A regulates MYC in CRC, we dissected MYC translation and stability dependent on CIP2A in CRC cell lines.

**Results:**

Knockdown of CIP2A reduced MYC protein levels without influencing MYC stability in CRC cell lines. Interfering with proteasomal degradation of MYC by usage of FBXW7-deficient cells or treatment with the proteasome inhibitor MG132 did not rescue the effect of CIP2A depletion on MYC protein levels. Whereas CIP2A knockdown had marginal influence on global protein synthesis, we could demonstrate that, by using different reporter constructs and cells expressing MYC mRNA with or without flanking UTR, CIP2A regulates MYC translation. This interaction is mainly conducted by the MYC 5′UTR.

**Conclusions:**

Thus, instead of targeting MYC protein stability as reported for other tissue types before, CIP2A specifically regulates MYC mRNA translation in CRC but has only slight effects on global mRNA translation. In conclusion, we propose as novel mechanism that CIP2A regulates MYC on a translational level rather than affecting MYC protein stability in CRC.

**Electronic supplementary material:**

The online version of this article (10.1007/s00384-020-03772-y) contains supplementary material, which is available to authorized users.

## Introduction

With more than 1.2 million newly diagnosed cases per year, colorectal cancer (CRC) is the most common gastrointestinal malignancy [1]. Sequence analysis shows that each tumor genome carries multiple mutations deregulating major signaling pathways that control growth and survival of colon epithelial cells [2]. Despite the genomic heterogeneity, enhanced MYC expression is universally observed in colon cancers. Gene expression analyses show that a signature of activated and repressed MYC target genes is present in a vast majority of CRC [2]. Deletion of the *MYC* gene ablates tumorigenesis in mouse models that faithfully mimic the human disease [3]. Collectively, these data argue that targeting MYC might achieve significant therapeutic efficacy in CRC.

Besides its transcriptional overexpression, MYC mRNA translation and stability are enhanced in cancer [4]. One major post-transcriptional regulator of MYC protein is the cancerous inhibitor of protein phosphatase 2A (CIP2A), which was identified as a human oncoprotein [5]. CIP2A is overexpressed in human tumor entities including CRC, gastric cancer, head and neck squamous cell carcinoma, breast cancer, prostate cancer, and lymphoma [6–8]. Enhanced expression of CIP2A correlates with reduced survival and serves as an independent negative predictive marker for overall and disease-free survival [9–14]. Several exogenous cancer-inducing factors, like *Helicobacter pylori* and papilloma virus 16 E7, upregulate the expression of CIP2A in host tissue which may be critical for their oncogenic activity [15, 16].

Consistent with CIP2A’s role as an inhibitor of the ubiquitous serine/threonine phosphatase PP2A which regulates the activity of numerous cellular signaling pathways [17], CIP2A has been shown to regulate the phosphorylation and activity of AKT, DapK, E2F1, MYC, and mTORC [13, 18, 19]. We and others have shown that on a molecular level, CIP2A regulates MYC post-transcriptionally due to several mechanisms. First, PP2A has a critical role in the turnover of MYC, since PP2A dephosphorylates MYC at serine 62 (S62). Dephosphorylation at S62 is required for ubiquitination of MYC by the ubiquitin ligase FBXW7 and therefore initiates degradation of MYC [6, 7]. Overexpression of CIP2A inhibits PP2A activity and thereby leads to MYC stabilization. Second, it has been shown that CIP2A specifically stabilizes pS62-MYC by interaction with the Lamin A/C complex in the nucleus [20]. Consequently, overexpression of CIP2A induces immortalization and malignant transformation of human cells.

Here, we show that CIP2A regulates MYC protein expression post-transcriptionally in CRC and that this regulation occurs via MYC mRNA translation rather than MYC stability.

## Results

**Fig. 1:**
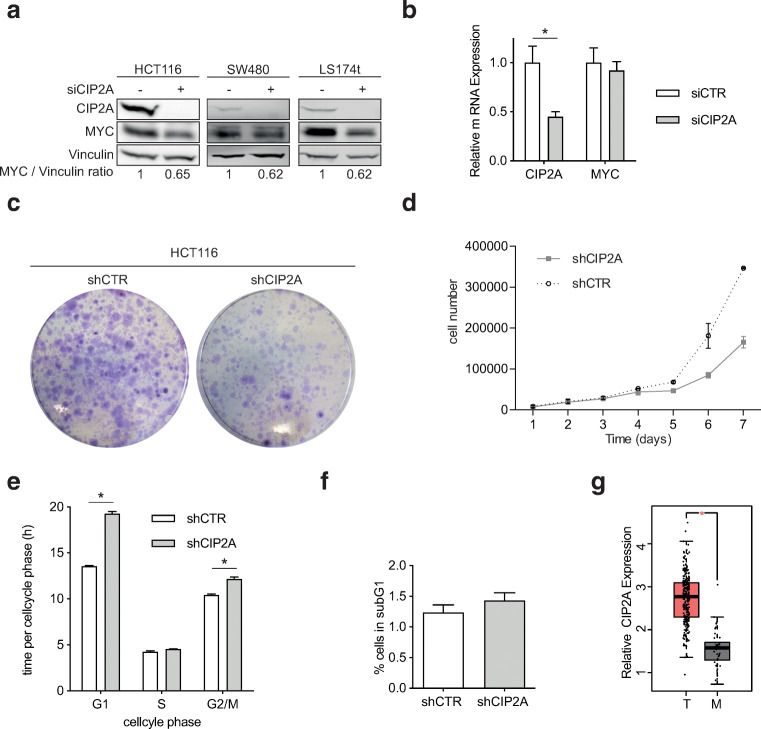
Depletion of CIP2A regulates MYC protein and cell proliferation in CRC cell lines. **a** Western blot analysis of CIP2A and MYC protein expression in HCT116, SW480, and LS174t cells transfected with CIP2A or CTR siRNA for 72 h. Data are representative of three independent experiments. **b** RT-QPCR analysis of CIP2A and MYC mRNA expression in HCT116 72 h after transfection with siRNA targeting CIP2A (mean of three independent biological experiments, error bars represent +/− SD). **c** Crystal violet staining of HCT116 cells stably infected with CIP2A or CTR shRNA after 7 days in culture. **d** Cell number of HCT116 cells stably infected with CIP2A or CTR shRNA during 7 days in culture (mean of three independent biological experiments, error bars represent +/− SD). **e** Time per cell cycle phase of HCT116 cells after CIP2A knockdown (* *p* < 0.001) (mean of three independent biological experiments, error bars represent +/− SD). **f** Fraction of cells in subG1 phase after knockdown of CIP2A (mean of three independent biological experiments, error bars represent +/− SD). **g** Expression of CIP2A in CRC (T) or corresponding mucosa (M) (* *p* < 0.01)

To analyze the impact of CIP2A on MYC protein expression and to recapitulate already published data in the setting of CRC, CIP2A expression was downregulated via siRNA in three CRC cell lines (HCT116, SW480, and LS174t). CIP2A knockdown led to substantial reductions in MYC protein levels in all three cell lines (Fig. 1a). This was due to post-transcriptional regulation of MYC protein levels, as no changes in *MYC* mRNA expression were detected (Fig. 1b). To rule out off-target effects of the used siRNA pool, HCT116 cells were transfected with three independent siRNAs side-by-side. Two of the three tested siRNAs led to a strong downregulation of CIP2A on mRNA and protein level as well as a reduced expression of MYC on protein, but not on mRNA level (Supplementary Fig. 1A, B). In contrast, one siRNA (#2) only leads to a slight knockdown of CIP2A and does not reduce MYC protein expression. In conclusion, this data argues that knockdown of CIP2A regulates MYC on protein level.

To determine long-term effects of CIP2A depletion on cell proliferation, HCT116 cells were stably infected with an shRNA targeting CIP2A. shRNA-mediated knockdown of CIP2A led to a growth defect in these cells, furthermore validating the results of siRNA-mediated depletion of CIP2A (Fig. 1c, d). Flow cytometry analysis (FACS) of propidium-iodide stained cells upon CIP2A knockdown revealed an accumulation of cells in G1 phase of the cell cycle, but the subG1 phase remained unaltered (Fig. 1e, f). This is comparable to a cell cycle arrest observed after reduction of MYC levels in other settings [21–23]. To validate the overexpression of CIP2A in CRC, we analyzed expression data in 275 CRC and 41 mucosa samples from ciBioPortal (https://www.cbioportal.org). This clearly demonstrated a strong upregulation of CIP2A in cancer tissue (Fig. 1g).

**Fig. 2:**
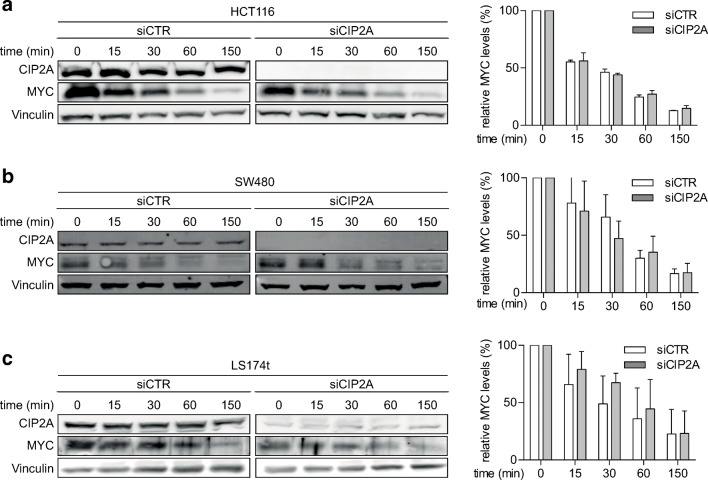
Depletion of CIP2A does not alter MYC protein stability in CRC cells. Immunoblots documenting MYC protein stability. Cells were incubated with cycloheximide (50 μg/ml) for the indicated time. **a** HCT116 left panel, immunoblot; right panel, quantification of MYC to vinculin ratio. **b** SW480 left panel, immunoblot; right panel, quantification of MYC to vinculin ratio. **c** LS174t left panel, immunoblot; right panel, quantification of MYC to vinculin ratio. Immunoblots are representative of three independent experiments. Quantifications show mean of three independent biological experiments; error bars represent +/− SD

In many cell lines, MYC proteins turn over with a half-life of approximately 20 min [24]. To determine the stability of MYC in CRC cell lines dependent on CIP2A, we treated cells with cycloheximide to inhibit new protein synthesis and measured the amount of remaining MYC protein by immunoblotting at several time points after treatment (Fig. 2a–c). MYC turned over with a half-life of approximately 30 min in all three CRC cell lines. Surprisingly, knockdown of CIP2A by siRNA did not affect MYC protein stability in any of the cell lines (Fig. 2a–c).

**Fig. 3:**
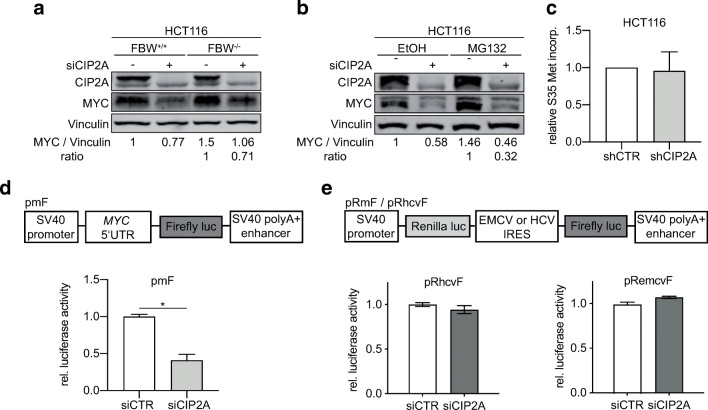
Inhibition of proteasomal degradation does not rescue MYC protein expression after CIP2A knockdown. **a** Western blot analysis of CIP2A and MYC protein expression in FBXW7-proficient (FBXW7^+/+^) and -deficient (FBXW7^−/−^) HCT116 cells. Data are representative of three independent experiments. **b** Western blot analysis of CIP2A and MYC protein expression in HCT116 cells treated with the proteasomal inhibitor MG132 (50 μg/ml) or solvent control (ETOH). Data are representative of three independent experiments. **c** Incorporation of S35-labeled methionine in HCT116 cells after knockdown of CIP2A (mean of three independent biological experiments, error bars represent +/− SD). **d** HCT116 cells were transfected with MYC 5′UTR bearing pmF luciferase reporter and transfected with siCIP2A or siCTR (mean of three independent biological experiments, error bars represent +/− SD; *p < 0.01). **e** HCT116 cells were transfected with bicistronic luciferase reporter vectors containing the HCV or EMCV IRES-element and transfected with siCIP2A or siCTR (mean of three independent biological experiments, error bars represent +/− SD)

MYC stability and phosphorylation is regulated by growth factor-dependent ERK and PI3K signaling pathways. Phosphorylation of MYC at serine 62 by ERK is necessary for recognition and subsequent phosphorylation at threonine 58 by GSK3β. Phosphorylated T58 is recognized by PIN1, as prerequisite for PP2A-dependent dephosphorylation of MYC proteins at S62 [4, 25, 26]. MYC protein, exclusively phosphorylated at T58, is recognized and ubiquitinated by SCF-FBXW7 and thereby primed for proteasomal degradation [4]. If CIP2A does not influence MYC protein stability, as suggested by the results of the cycloheximide assay, knockdown of CIP2A should still reduce MYC protein levels even if protein degradation is blocked. To evaluate the impact of CIP2A-dependent MYC expression and FBXW7-dependent MYC degradation, CIP2A expression was downregulated via siRNA transfection in both HCT116 FBXW7^+/+^ and HCT116 FBXW7^−/−^ cells (Fig. 3a). As expected, FBXW7-deficient cells showed per se higher MYC protein levels compared to FBXW7-proficient cells. Knockdown of CIP2A led to a comparable reduction of MYC protein in both conditions. To rule out the possibility that CIP2A primes MYC for another ubiquitin E3 ligase, overall protein degradation was inhibited by treating cells with the proteasome inhibitor MG132. MG132 treatment led to an expected accumulation of MYC and, to a lower extent, of CIP2A (Fig. 3b). In both MG132-treated and untreated cells, knockdown of CIP2A led to a comparable downregulation of MYC protein levels (Fig. 3b). In conclusion, our data suggest that CIP2A does affect neither MYC transcription nor MYC protein stability in CRC, but that CIP2A rather influences translation of MYC.

To test whether CIP2A has an impact on global protein synthesis, HCT116 cells were starved for 20 min in methionine-free media. After that, incorporation of S^35^-labeled methionine within 1 h was measured (Fig. 3c). Knockdown of CIP2A did not show any effect on global protein synthesis. To clarify whether CIP2A directly influences MYC translation, a reporter plasmid, carrying the *MYC* 5′UTR ahead of the coding sequence of a firefly luciferase (pmF), was used. Knockdown of CIP2A led to a 60% decrease in relative luciferase activity, pointing towards a direct influence of CIP2A on MYC translation (Fig. 3d). To test whether CIP2A knockdown affects the translation from other structured 5′UTRs, we used bicistronic vectors containing the IRES element of the EMCV or HCV virus. In both conditions, knockdown of CIP2A does not alter luciferase activity (Fig. 3e). In summary, we concluded that CIP2A regulates MYC on a translational level in CRC.

**Fig. 4:**
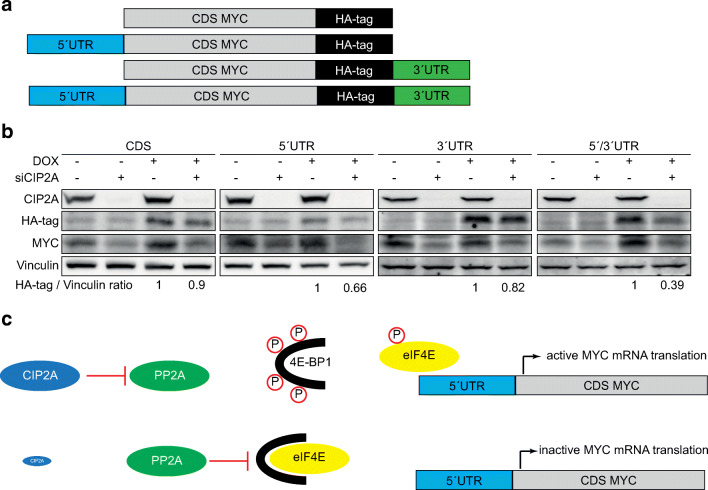
CIP2A regulates MYC mRNA translation initiation. **a** Schematic illustration describing the MYC constructs used in B. **b** Western blot analysis of CIP2A, MYC, and HA-tag expression in HCT116 after knockdown of CIP2A. Data are representative of three independent experiments. Every construct was evaluated on separate gels. **c** Summary of suggested molecular mechanism.

mRNA translation can be regulated by several upstream pathways, e.g., mTOR signaling, or via direct regulation of translation initiation or elongation factors [27]. We have previously shown that CIP2A does not regulate mTOR signaling in CRC [7]. To evaluate whether CIP2A affects MYC translation initiation or elongation, we used HCT116 cells with doxycycline-inducible MYC constructs expressing the MYC coding sequence (CDS) with or without 5′/3′UTRs with an HA-tag to distinguish between endogenous and exogenous MYC [22, 28] (Fig. 4a). Whereas endogenous MYC protein was reduced by CIP2A knockdown, MYC constructs expressing the MYC CDS or MYC CDS + 3′UTR remained mainly unaffected by CIP2A knockdown (Fig. 4b). However, the construct expressing the MYC CDS + 5′UTR was robustly downregulated to a ratio of 0.66 after CIP2A knockdown. The most prominent effect on MYC protein reduction after CIP2A knockdown was observed with the construct expressing the MYC 5′UTR + CDS + 3′UTR (ratio of 0.39, Fig. 4b). In conclusion, these data show that CIP2A regulates MYC translation initiation rather than elongation, as the expression of the MYC CDS is unaffected.

## Discussion

Deregulated and enhanced expression of MYC is a driver of colorectal tumorigenesis, necessitating strategies to inhibit MYC function or expression for tumor therapy. We and others have shown that CIP2A regulates MYC, is essential for tumor growth, tissue regeneration, and thus may open a therapeutic window for targeting tumors [6, 7, 10, 18, 20, 29].

It has been clearly demonstrated that CIP2A reduces MYC expression post-transcriptionally. For example, mouse embryonic fibroblasts (MEF) isolated from CIP2A-deficient (CIP2A^HOZ^) mice show the same amount of *MYC* mRNA as wild-type mice, but harbor lower MYC protein levels after serum stimulation [20]. In line with this are our own published results that CIP2A does not influence *MYC* mRNA expression arguing that MYC is regulated post-transcriptionally by CIP2A [7]. So far, on a molecular level, it was shown that CIP2A regulates the stability of MYC, mainly by stabilizing pS62-MYC. Here, we describe a novel mechanism of regulation of MYC translation by the oncoprotein CIP2A via the 5′UTR of the *MYC* mRNA. It remains elusive which factor drives MYC translation in tumors and is regulated by CIP2A. Still, our experiments showing that MG132 treatment does not rescue downregulation of MYC protein upon CIP2A knockdown clearly argue against a regulation of MYC stability by CIP2A in CRC.

There are several possibilities how MYC translation can be regulated via CIP2A. First, PP2A can mediate regulation of upstream pathways like mTOR signaling which has been proposed previously [30]. This is unlikely for CRC, as we have already demonstrated that PI3K/AKT/mTOR signaling is not affected by CIP2A [7]. Second, CIP2A could directly regulate the function or activity of translation factors. The process of mRNA translation is divided in three regulatory parts, initiation, elongation, and termination [27]. Regarding elongation, our experiments do not show an effect on MYC CDS expression after CIP2A depletion, arguing that CIP2A does not act via the regulation of elongation of the MYC mRNA. In contrast, CIP2A influences the 5′UTR of MYC as demonstrated in our experiments by regulation of firefly luciferase expression as well as exogenous constructs carrying MYC CDS + 5′UTR. Whereas global protein synthesis is unaltered or only slightly reduced, which could be explained by the reduced MYC protein levels and cell proliferation, the firefly luciferase activity and MYC protein levels are decreased to a much greater extent. These results strongly support the notion that CIP2A regulates MYC translation initiation. Several oncogenes, like *MYC*, *SRC*, and *VEGF*, harbor complex structures in their 5′UTR, such as G-quadruplexes structures or IRES elements, enhancing their translation in specific situations like tumor development [31]. The possibility that CIP2A regulates MYC translation via such a structure is promoted by our results showing a difference in translation efficacy dependent on the 5′UTR of MYC. The exact translation initiation factor which regulates CIP2A-dependent MYC translation in CRC is not known so far.

It has been shown that CIP2A regulates the phosphorylation of target proteins via inhibition of PP2A-mediated dephosphorylation [32]. So far, several studies have demonstrated that PP2A, besides the regulation of MYC via its dephosphorylation, is also able to function in mRNA translation. In lung fibroblasts, PP2A regulates 4E-BP1, the eIF4E inhibitor, by preventing its degradation and by this reducing CAP-dependent translation [33]. In addition to 4E-BP1, PP2A directly influences the activity of eIF4E by dephosphorylating MNK1 and eIF4E at their activating phosphorylation sites, thereby reducing MYC and MCL-1 expression [34]. It is therefore possible that CIP2A regulates MYC mRNA translation indirectly via regulating the activity of the CAP-binding complex (Fig. 4c). Consistent with this hypothesis, it has been shown that the majority of CIP2A is localized in the cytoplasm rather than in the nucleus where it regulates the association of MYC with the Lamin A/C complex and pS62-MYC stability [20].

Mounting evidence indicates that protein synthesis is deregulated in tumors in a way that in total, more and different mRNAs are translated compared to normal tissue [28, 35, 36]. In line with this, we show here that depletion of CIP2A specifically decreases translation of MYC.

In conclusion, we demonstrate a possible novel way of action of CIP2A controlling MYC expression specifically via regulation of MYC translation in CRC.

## Methods

### Cell lines and cell culture

HCT116, LS174t, and SW480 cells were purchased from American Type Culture Collection. All cell lines were authenticated via Short Tandem Repeat (STR) DNA analysis. HCT116 cells were cultured in DMEM, LS174t, and SW480 cells in RPMI-1640 medium supplemented with 10% heat-inactivated fetal calf serum (FCS) and 1% penicillin-streptomycin. Cells were cultured in an incubator at 37 °C, 95% humidity, and 5% CO_2_.

### Real-time quantitative reverse transcription-PCR analysis

mRNA expression was analyzed using real-time quantitative reverse transcription-PCR (RT-QPCR). Total cellular RNA was extracted from cells with RNeasy Minikit (Qiagen) according to the manufacturer’s instructions. Relative quantification, based on the fold difference, was calculated with the threshold cycle (Ct) method, expressed as 2^−ΔΔCt^.

### Immunoblot analysis

Cultured cells were rinsed three times with ice-cold PBS, harvested, and lysed directly in RIPA sample buffer for western blot analysis. Cell debris were removed by centrifugation at 12,000*g* for 10 min at 4 °C, and the supernatant was used as total protein lysate. For each sample, 10 μg of total protein lysate was subjected to a 10% sodium dodecyl sulfate-polyacrylamide gel electrophoresis (SDS-Page), followed by western blot analysis. Western blots were probed with antibodies against CIP2A (A301-454A; Bethyl Laboratories), MYC (C33, #42; Santa Cruz; or Y69 # ab32072 Abcam), β-actin (AC-15/A5441; Sigma), vinculin (V9131; Sigma), and HA-tag (HA-Tag; C29F4 #3724S cell signaling). All antibodies were used according to the manufacturer’s instructions. The blots were visualized with secondary antibodies (GE Healthcare) against mouse (NA9310) or rabbit (NA9340) primary antibodies.

### siRNA transfection

On-target plus SMART pool (Horizon Discovery) siRNA to target CIP2A (L-014135-01-005) and a control siRNA (D-001810-10-05) were used for silencing gene expression. Cells were transfected with siRNA pools (final concentration 100 nM) using RNAiMax kit (Invitrogen) according to the manufacturer’s protocol. Cells were harvested 72 h after transfection.

### shRNA and lentivirus

shRNA sequences targeting CIP2A were cloned into a lentiviral pGIPZ vector according to the manufacturer’s protocol. HEK293t cells were transfected together with packaging plasmids. After 48 h and 72 h, supernatants containing the virus were collected and filtered. Colon cancer cell lines were infected with the shCIP2A-expressing lentivirus or non-specific shCTR, and 24 h post-transduction infected cells were selected with puromycin.

### Colony formation assay

2.5 × 10^3^ cells infected with shCIP2A-expressing lentivirus or shCTR were plated on 6-well plates. Colonies were stained with 0.5 % cystal violet in 20% ethanol after 7 days in culture.

### Luciferase assay

Plasmids and protocol for luciferase assay have previously been described in [37].

### S^35^-Methionine incorporation assay

Global translation was measured by S^35^-methionine incorporation assay. Cells were seeded in 6-well plates and treated as indicated. To deplete the intracellular pool of methionine, cells were washed with PBS and incubated in RPMI medium lacking methionine for 20 min. Cells treated with 50 μg/ml cycloheximide were used as a control for translation inhibition. Cells were pulsed with 1 μCi/ml S^35^-methionine for 1 h at 37 °C. To remove unincorporated S^35^-methionine, cells were washed 5 times with ice-cold PBS prior to precipitation with 10% TCA for 20 min. Cells were lysed with 300 μl 0.2 M NaOH and collected in a 1.5-ml tube. Fifty-microliter cell lysate was added to 3-ml scintillation solution in a scintillation tube and vortexed for 20 s. Radioactive counts were measured by scintillation counting. Protein content in cell lysates was quantified using BCA reagent and used to normalize the radioactive counts.

## Electronic supplementary material

Supplementary Figure 1: (A) Western blot analysis of CIP2A and MYC protein expression in HCT116 cells transfected with three independent siRNAs targeting CIP2A or siCTR for 72 h. Data are representative of three independent experiments. (B) RT-qPCR analysis of CIP2A and MYC mRNA expression in HCT116 72 h after transfection with three different siRNAs targeting CIP2A. (PDF 87 kb)
