# Supplementary figures and images for: CIP2A regulates MYC translation (via its 5′UTR) in colorectal cancer
Source: Int J Colorectal Dis. 2020 Oct 19;36(5):911–8. doi: 10.1007/s00384-020-03772-y (PMC8178152; doi:10.1007/s00384-020-03772-y)

Supple. Figure 1

A

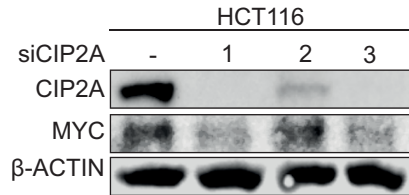

B

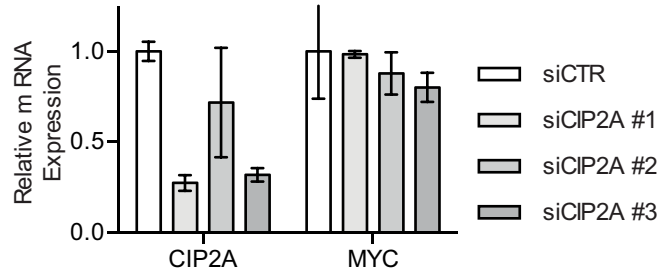

Supplement: Supplementary file 1 — (A) Western blot analysis of CIP2A and MYC protein expression in HCT116 cells transfected with three independent siRNAs targeting CIP2A or siCTR for 72 h. Data are representative of three independent experiments. (B) RT-qPCR analysis of CIP2A and MYC mRNA expression in HCT116 72 h after transfection with three different siRNAs targeting CIP2A. (PDF 87 kb) [file 384_2020_3772_MOESM1_ESM.pdf]
